# Unphosphorylated STAT3 in heterochromatin formation and tumor suppression in lung cancer

**DOI:** 10.1186/s12885-020-6649-2

**Published:** 2020-02-22

**Authors:** Pranabananda Dutta, Lin Zhang, Huijun Zhang, Qin Peng, Phillippe R. Montgrain, Yingxiao Wang, Yuanlin Song, Jinghong Li, Willis X. Li

**Affiliations:** 1 Department of Medicine, University of California San Diego, La Jolla, CA 92093 USA; 2 Department of Pulmonary Medicine, Shanghai Respiratory Research Institute, Zhongshan Hospital, Fudan University, Shanghai, China; 3 Department of Bioengineering, Institute of Engineering in Medicine, University of California San Diego, La Jolla, CA 92093 USA; 4 Veterans Affairs San Diego Healthcare System, San Diego, CA CA92037 USA

**Keywords:** USTAT3, Heterochromatin, JAK/STAT, Lung cancer, FRET

## Abstract

**Background:**

Aberrant JAK/STAT activation has been detected in many types of human cancers. The role of JAK/STAT activation in cancer has been mostly attributed to direct transcriptional regulation of target genes by phosphorylated STAT (pSTAT), while the unphosphorylated STAT (uSTAT) is believed to be dormant and reside in the cytoplasm. However, several studies have shown that uSTATs can be found in the nucleus. In addition, it has been shown that tissue-specific loss of STAT3 or STAT5 in mice promotes cancer growth in certain tissues, and thus these STAT proteins can act as tumor suppressors. However, no unifying mechanism has been shown for the tumor suppressor function of STATs to date. We have previously demonstrated a non-canonical mode of JAK/STAT signaling for *Drosophila* STAT and human STAT5A, where a fraction of uSTAT is in the nucleus and associated with Heterochromatin Protein 1 (HP1); STAT activation (by phosphorylation) causes its dispersal, leading to HP1 delocalization and heterochromatin loss.

**Methods:**

We used a combination of imaging, cell biological assays, and mouse xenografts to investigate the role of STAT3 in lung cancer development.

**Results:**

We found that uSTAT3 has a function in promoting heterochromatin formation in lung cancer cells, suppressing cell proliferation in vitro, and suppressing tumor growth in mouse xenografts.

**Conclusions:**

Thus, uSTAT3 possesses noncanonical function in promoting heterochromatin formation, and the tumor suppressor function of STAT3 is likely attributable to the heterochromatin-promoting activity of uSTAT3 in the non-canonical JAK/STAT pathway.

## Background

In the canonical JAK/STAT signaling pathway, activated JAK phosphorylates STAT at a tyrosine residue around a.a.700, and the resulting phosphorylated STAT (pSTAT) dimerizes and translocates to the nucleus, where it functions as a transcription factor, while the dormant unphosphorylated STAT (uSTAT) in the cytoplasm has no significant functions. However, our previously research using *Drosophila* STAT92E and human STAT5A has demonstrated a non-canonical JAK/STAT signaling, in which uSTAT is capable of associating with HP1 and stabilizing heterochromatin [1, 2]. JAK activation can increase pSTAT and decrease uSTAT, thus causing heterochromatin instability [3, 4]. Other groups have shown that human JAK2 activation reduces heterochromatin in leukemia and stem cells [5–8].

Many groups have reported that uSTATs can translocate into and prominently exist in the nucleus in various mammalian cells at quiescence, when STAT proteins are not phosphorylated [9–16]. Specifically, it has been shown that STAT3 maintains a prominent nuclear presence independent of its tyrosine phosphorylation status in several mammalian cell lines [12, 13, 16], and that uSTAT5 similarly is detected in the nucleus of serum-starved unstimulated cells, where STATs are not phosphorylated [11, 17, 18]. Further, uSTAT1, 3, and 5 can bind to DNA [18–21] and to regulate gene transcription [9, 13, 14, 18].

Our previous work has shown that the STAT-HP1 interaction is mechanistically and functionally conserved in human cells for STAT5A [2], which is most homologous to *Drosophila* STAT92E [22, 23]. We have shown that both endogenous STAT5A and transfected uSTAT5A (or STAT5A^Y694F^) are prominently present in the nucleus of cultured human cells [2]. This observation is consistent with reports by other groups (see Fig. 1a in ref. [11]; Fig. 5A in [17]; Fig. 1 in ref. [18]). In addition, uSTAT5A physically interacts with HP1α via an HP1-binding motif, PxVxI, present in STAT proteins [1, 2]. It has been shown that uSTAT5 in the nucleus directly binds to and represses differentiation genes in hematopoietic progenitor cells [18]. Thus, the “textbook” version of JAK/STAT signaling needs revision; uSTATs are not simply “latent cytoplasmic proteins” but constantly shuttle into the nucleus [15, 24], where they may function to regulate gene transcription [14, 18] and promote heterochromatin stability [2, 4, 25].

While many groups have demonstrated nuclear localization of uSTATs, their nuclear functions are nonetheless less clear. Although it is reported that uSTAT3 can activate gene expression [14], genomic studies have shown that uSTAT5 is mainly involved in gene repression, that activation of the JAK/STAT pathway causes genome-wide redistribution of chromatin-bound STAT5 to traditional STAT transcriptional targets, due to conversion of uSTAT5 to pSTAT5, and that either STAT5 activation or its depletion causes derepression of differentiation genes [18]. This latter finding is consistent with our studies of STAT5A [2]. We have shown that uSTAT5A functions strikingly similar to HP1α in gene repression, and that many of the genes repressed by uSTAT5A and HP1α in common are overexpressed in colon cancer [2]. Importantly, these same genes increase their expression when endogenous STAT5A or HP1α is knocked down, suggesting that endogenous uSTAT5A and HP1α are involved in repressing these genes possibly via heterochromatin formation.

Heterochromatin is important for chromosomal compaction and transcriptional silencing as well as for genome stability, animal longevity, and tumor suppression [26–28]. Cellular differentiation is associated with increases in heterochromatin levels [29–32]. The reverse of this process, i.e., dedifferentiation, is a hallmark of cancer development [33]. Loss of heterochromatin and derepression of satellite repeats is found in many cancers [34]. Tumorigenesis occurs only in cells that have decreased levels of heterochromatin or are unable to form new heterochromatin [35, 36]. Heterochromatin is marked by di- or tri-methylated lys9 on histone H3 (H3K9me3), which provides docking site for HP1, both of which are hallmarks of heterochromatin [26–28]. It has been shown that Suv39h1, a H3K9-specific histone methyl transferase (HMT) and key component of heterochromatin, functions as a tumor suppressor whose loss permits lymphoma development in response to oncogenic Ras [35]. Consistent with these findings, we have shown that heterochromatin is essential for maintaining genome stability [37], and suppresses tumor growth [2].

Since the HP1-binding motif PxVxI is conserved in all STAT proteins including STAT3, we sought to investigate whether uSTAT3 can also bind HP1 and can thereby play a role in tumor suppression. Activation of STAT3 has been found more often than STAT5 or any other STATs in cancers. Thus, understanding the functions of STAT3 is important in cancer biology. Interestingly, it has been shown that loss of STAT3 or STAT5 promotes cancer growth in certain tissues, suggesting these STAT proteins might function as tumor suppressors in these situations [38–42]. On the other hand, several groups have shown that increasing uSTAT3 levels inhibits tumor growth [43, 44], an effect that has been attributed to its dominant-negative interference with transcriptional activation of pSTAT3. The apparently contradicting results regarding STAT3 in lung cancer are entirely consistent with our hypothesis that the tumor suppressor function of STAT3 stems from its noncanonical function in controlling heterochromatin, i.e., uSTAT3 rather than pSTAT3 suppresses tumors. Our hypothesis predicts that expressing uSTAT3 and STAT3 knockdown would have opposite effects on lung cancer growth.

To understand the biological functions of STAT3 in lung cancer and to determine whether noncanonical function of STAT3 plays a role in lung cancer development, we investigated the interaction between STAT3 and HP1α in a few non-small cell lung cancer (NSCLC) cell lines. We found that STAT3 and HP1α partially colocalize in the nucleus and might physically interact, and that uSTAT3 promotes heterochromatin formation and suppresses lung cancer cell proliferation in vitro and in vivo. These results suggest that the non-canonical functions of STAT3 operate in lung cancer cells, in which uSTAT3 plays a role in suppressing cancer growth.

## Results

### Nuclear localization of uSTAT3 and its physical interaction with HP1α

**Fig. 1:**
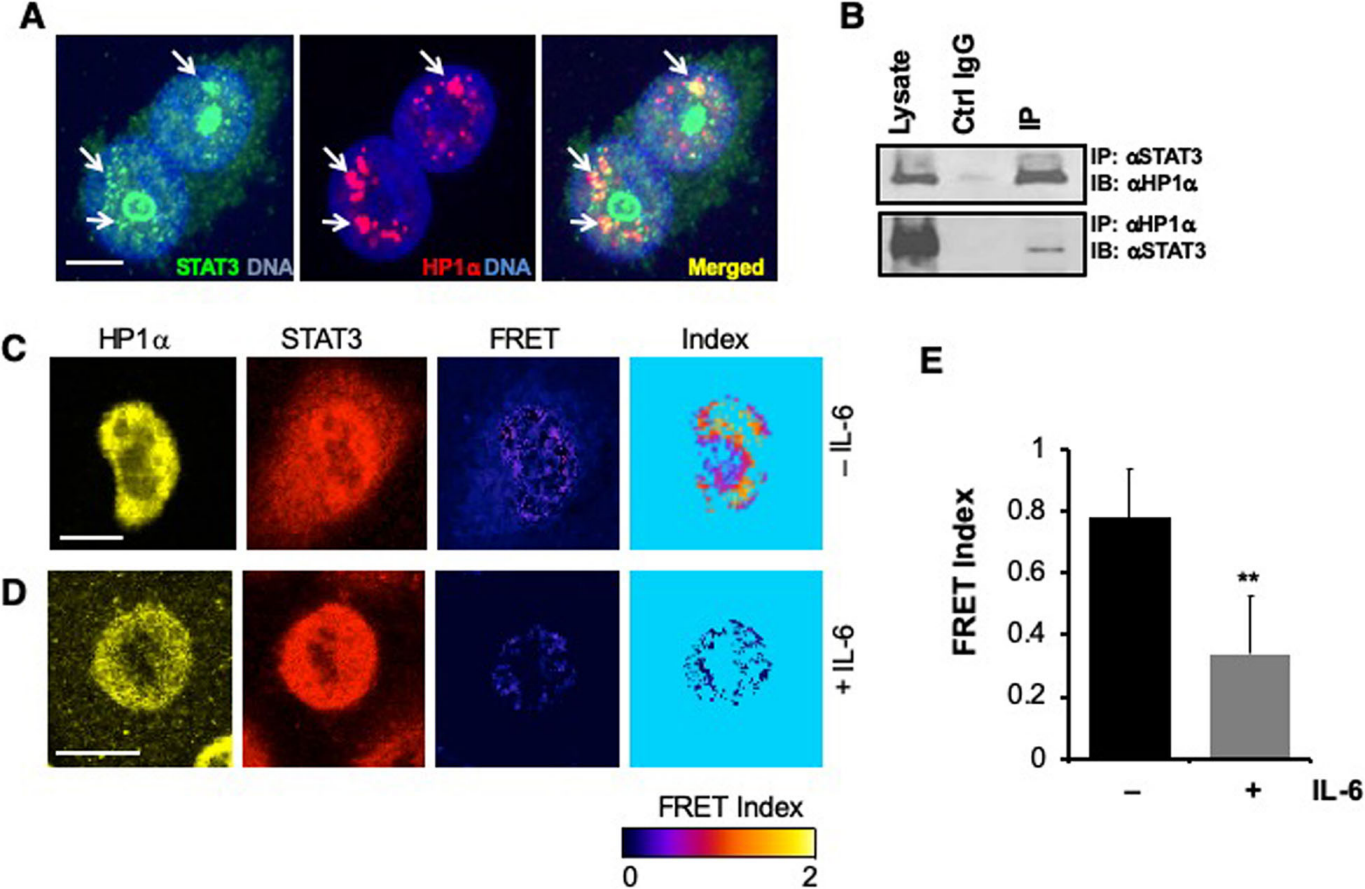
STAT3 and HP1α physically interact. **(a)** A549 cells were fixed and immunostained with anti-STAT3 (Green) and anti-HP1α (Red) and were imaged using confocal microscopy. A 1 μm optical section is shown to reveal colocalization in discrete regions (arrows). **(b)** A549 cell lysates were immunoprecipitated with anti-STAT3 (top panel) or anti-HP1α (lower panel) antibodies, the presence of HP1α and STAT3 in the immunoprecipitates were detected by Western blotting with the respective antibodies. **(c, d)** A549 cells were fixed and immunostained with anti-HP1α-Alexa488 (donor) to anti-STAT3-Alexa546 (acceptor). Donor and acceptor bleed through was corrected using donor and acceptor only samples. FRET was detected and processed using a Leica confocal microscope with built-in FRET software. Note that FRET is detected in the nucleus **(C),** that IL6 treatment reduced FRET efficiency **(d)**. **(e)** FRET efficiency was quantified as YFP/CFP fluorescence ratio for cells with or without IL-6 treatment. ** indicates *p* < 0.01 in unpaired Student’s t-test. Scale bars, 2 μm

As has been previously reported, uSTAT3 is predominantly detected in the nucleus of various types of serum-starved, unstimulated cells (e.g., Fig. 1 in [12]). We examined the subcellular localization of endogenous STAT3 and HP1α in in serum starved cells using several lines of lung cancer cells including A549, H226, H441, H460, H520, as well as HeLa and HEK293T cells. When observed with a laser confocal microscope in thin optical sections, we found that endogenous STAT3 in all these cell lines can be detected in the nucleus as unevenly distributed particulates, some of which are colocalized with HP1α **(**Fig. 1a**)**. Some of these cancer cell lines show very low or no detectable pSTAT3 under unstimulated conditions, as previously reported (e.g., A549) [44]. The pattern of nuclear STAT3 distribution in these cells does not correlate with its phosphorylation status, consistent with previous reports that STAT3 is prominently detected in the nucleus regardless of its phosphorylation status [12, 13, 16]. In addition, we found that endogenous STAT3 and HP1α co-immunoprecipitate **(**Fig. 1b**),** consistent with the idea that they may physically interact.

To investigate if the partially colocalized STAT3 and HP1α in the nucleus indeed physically interact, we employed fluorescence resonance energy transfer (FRET), which is based on transfer of energy from one fluorescent molecule (donor) to another (acceptor) only when the two are in close proximity (< 10 nm apart), which occurs when the two molecules directly interact [45, 46]. In order to investigate the proximity of unmodified endogenous STAT3 and HP1α in cells, we used fluorophore-conjugated secondary antibodies and FRET [46, 47] in fixed cells. We found high FRET efficiency from anti-HP1α-Alexa488 (donor) to anti-STAT3-Alexa546 (acceptor) only in discrete regions of the nucleus (Fig. 1c), suggesting that STAT3 and HP1α physically interact in these regions of the nucleus. Importantly, when treated with the cytokine IL6, which activates STAT3 by phosphorylation, increasing pSTAT3 and decreasing uSTAT3, we found that FRET efficiency was significantly reduced, suggesting a loss of STAT3-HP1α interaction (Fig. 1d, e). The FRET results suggest that the colocalized uSTAT3 and HP1α physically interacts.

### uSTAT3 promotes heterochromatin formation

**Fig. 2:**
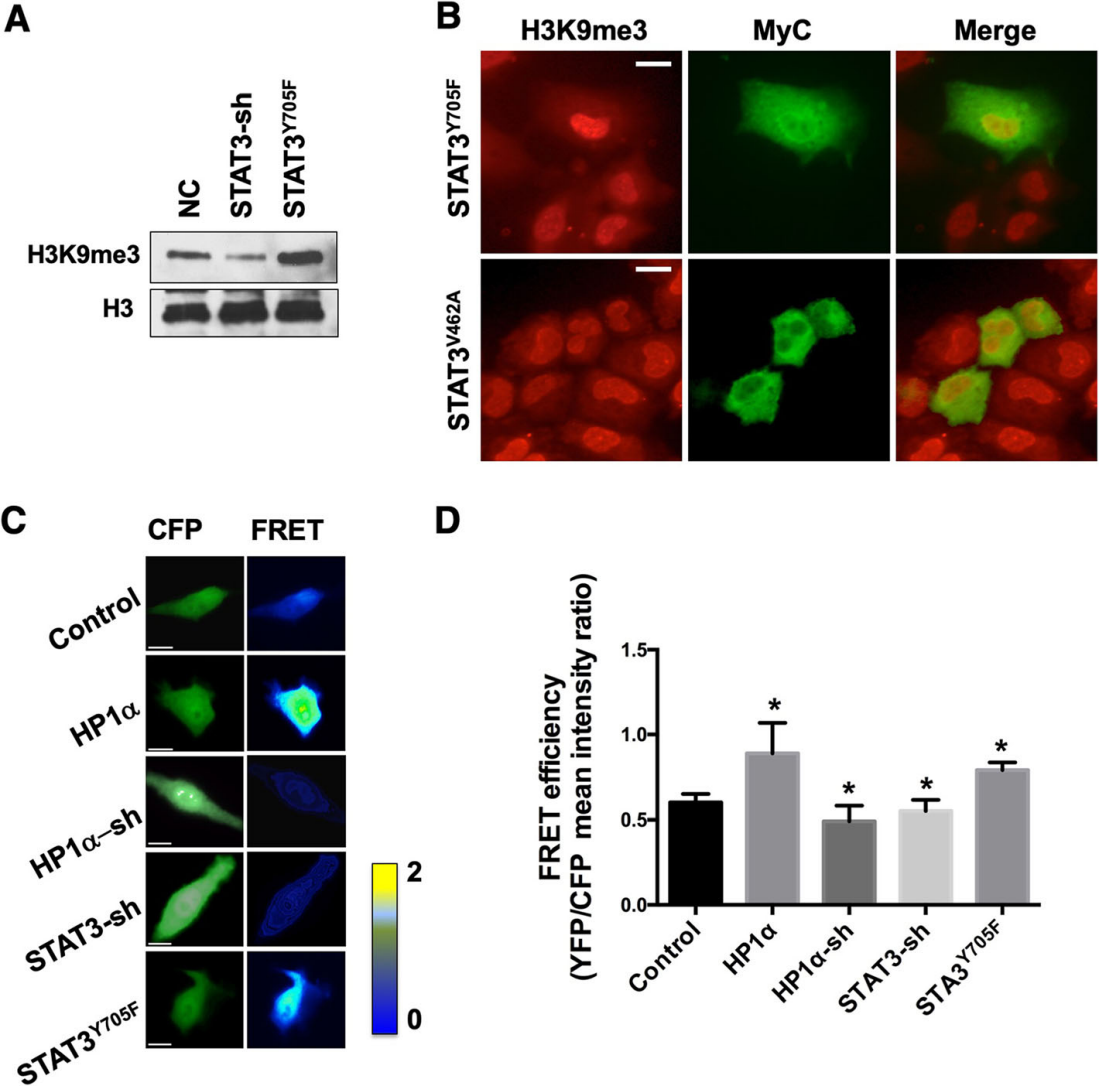
uSTAT3 promotes heterochromatin formation in lung cancer cells. **(a)** Total lysates of A549 cells stably transfected with the indicated DNA constructs were subjected to SDS-PAGE and blotted with anti-H3K9me3. Note that expressing STAT3^Y705F^ or shSTAT3 dramatically increased or moderately decreased H3K9me3 levels, respectively. sh: small hairpin RNAi; NC, non-targeting control. **(b)** A549 cells were transiently transfected with MyC-tagged STAT3^Y705F^ (upper) or STAT3^V462A^ (lower) were immunostained with anti-H3K9me3 (left) or Myc (middle). Note that STAT3^Y705F^, but not STAT3^V462A^, expression increased H3K9me3, when compared with untransfected neighboring cells. **(c,d)** A549 cells stably expressing the indicated STAT3 or HP1α transgenes were transfected with a heterochromatin FRET sensor, consisting of an HP1-H3 peptide with CFP and YFP. **(c)** Representative FRET images of cells with indicated transgene expression. **(d)** Mean FRET efficiency (YFP/CFP fluorescence ratio) with standard deviations was calculated for each group. * indicates *p* < 0.05 in Student’s t-test. Scale bars, 2 μm

To investigate if by binding to HP1α, uSTAT3 is able to promote heterochromatin formation, we carried out the following experiments. First, using Western blotting, we found that expressing an unphosphorylatable mutant STAT3 (STAT3^Y705F^), representing uSTAT3, causes a notable increase in the levels of the heterochromatin mark, H3K9me3, whereas depleting endogenous STAT3 leads to moderately reduced H3K9me3 levels (Fig. 2a). Second, by using immunostaining, we found that expressed STAT3^Y705F^ increased H3K9me3 levels, whereas expressing the non-HP1-interacting STAT3^V462A^ had no effects (Fig. 2b). Third, we employed a FRET-based heterochromatin sensor to investigate the effects of HP1α and uSTAT3 on global heterochromatin levels. The heterochromatin FRET sensor consists of peptides of H3 and HP1 fused to YFP and CFP, respectively, which has been previously used as a robust readout of H3K9me3 levels when expressed by transfection [48–50]. We found that, as expected, altering HP1α levels, by overexpressing HP1α or expressing a small hairpin (sh) HP1α RNA, affect global heterochromatin levels, as indicated by the FRET sensor (Fig. 2c, d). Importantly, we found that expressing STAT3^Y705F^ increased, whereas knocking down STAT3 decreased global heterochromatin levels in A549 cells (Fig. 2c, d). A hallmark of non-canonical STAT function is that expressing unphosphorylatable mutant STAT has the opposite effect to knocking down STAT, whereas in the canonical pathway, they have the same effect. These results suggest that STAT3 also has non-canonical function in regulating heterochromatin globally.

**Fig. 3:**
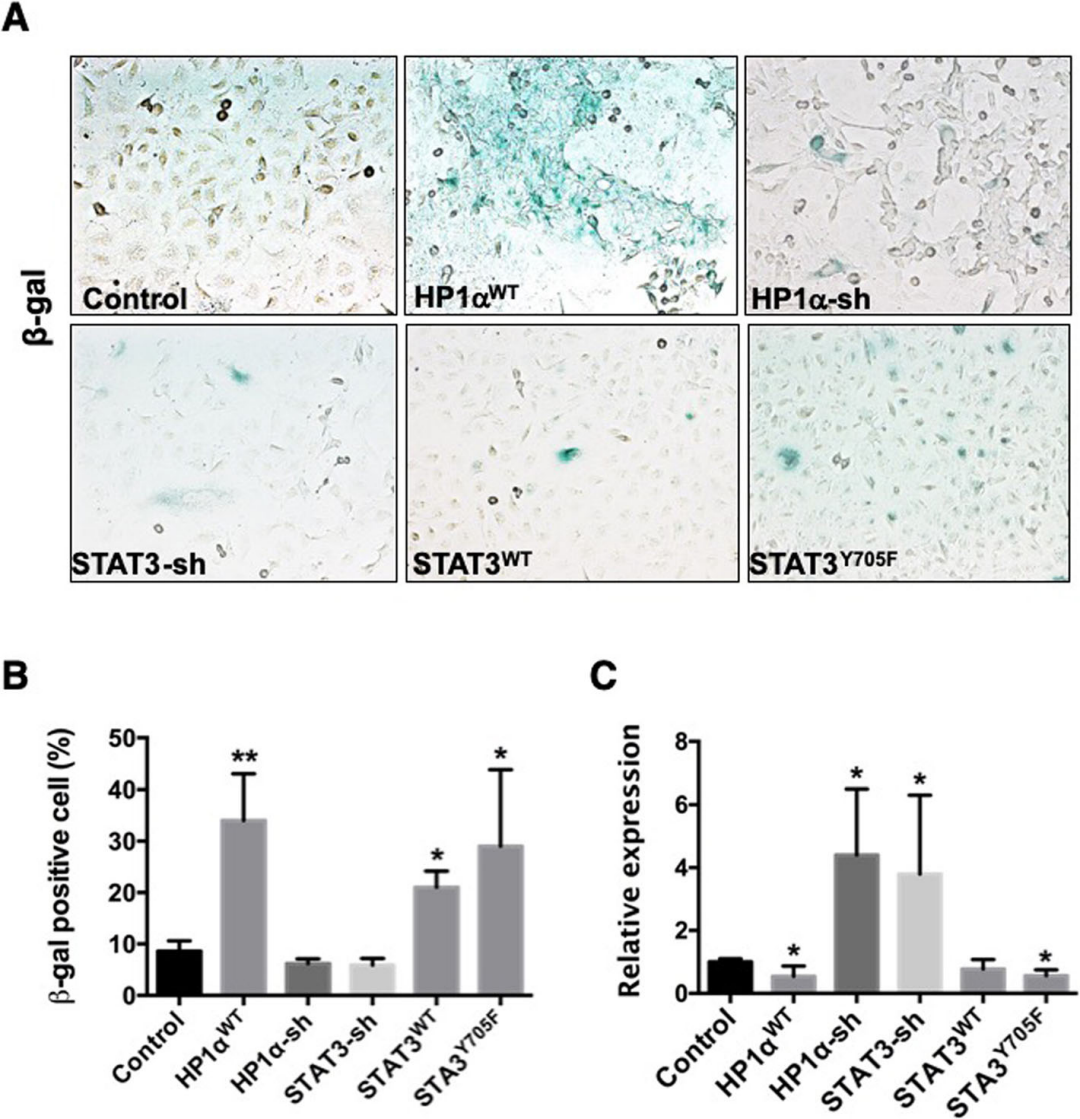
STAT3 and HP1α affect cellular senescence and major satellite transcription. (**a**) Representative images are shown of A549 cells stably expressing the indicated STAT3 or HP1α transgenes assayed for senescence-associated beta-galactosidase (SA-β-gal; blue) at pH 6.0 [53]. Control cells were parent A549 cells without transgene expression. (**b**) Cell senescence was quantified by measuring senescence-associated β-Gal staining at an absorbance of 405 nm. **(c)** Total RNA was isolated from A549 cells stably expressing the indicated transgene, and were quantified for major satellite transcripts using qRT-PCR. GAPDH gene transcript was used as the internal control for expression. The mean value ± s.d. (standard deviation) was calculated in each group. * P < 0.05, ** *P* < 0.01

To evaluate the changes in cellular phenotypes from altering heterochromatin, we carried out the following two experiments. First, we investigated cellular senescence, a state of irreversible cell cycle arrest associated with formation of specialized facultative heterochromatin, which is hypothesized to repress proliferation-promoting genes and suppress tumor development [35, 51]. Senescent cells are commonly detected by histochemical staining for senescence-associated β-galactosidase (SA-βgal) [52]. Using stable A549 cells, we found that expressing uSTAT3 or HP1α led to more senescent cells, whereas knocking STAT3 or HP1α resulted in fewer senescent cells (Fig. 3a, b). Second, heterochromatin is known to repress transcription from satellite repeats, whose derepression is found in many cancers [34]. Using quantitative real-time polymerase chain reaction (qRT-PCR), we found that uSTAT3 and HP1α repressed major satellite transcripts, whereas knocking down endogenous STAT3 or HP1α caused a 4-fold increase in major satellite transcripts (Fig. 3c).

### uSTAT3 and HP1α affects lung cancer cell growth in vitro and in vivo

**Fig. 4:**
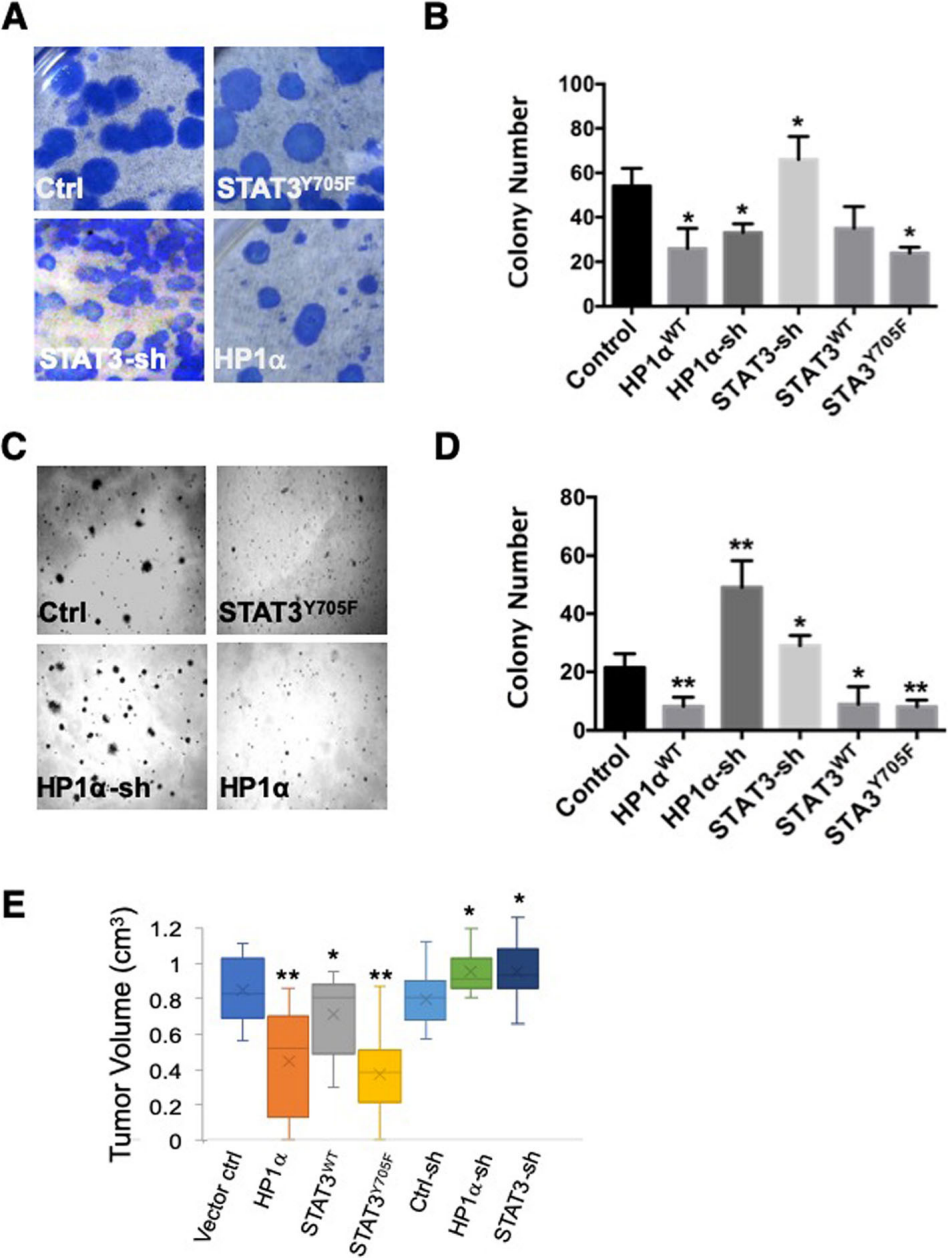
STAT3 and HP1α affect lung cancer cell growth in vitro. A549 cells stably expressing the indicated STAT3 or HP1α transgenes were subjected to clonogenic assay (**a**, **b**) and soft-agar assay (**c, d**). Represented images are shown. Colony numbers are presented as the mean value ± s.d. * indicates p < 0.05, ** *P* < 0.01, in Student’s t-test. **(e)** Box plots of tumor volumes 4 weeks after subcutaneous injection of A549 cells stably expressing the indicated constructs are shown. Each box shows the range of the second and third quartiles of tumor volumes. The “X” in the box indicates the median tumor volume. The bars (“whiskers”) represent the largest and smallest tumors. Six injections (*n* = 6) were done for each of the indicated transgene and cell line combination. n represents the number of injections for the indicated cell line. * and ** indicate p < 0.05 and *p* < 0.01 (Student’s t-test), respectively, when compared with the vector control or with control RNAi construct

To investigate whether uSTAT3 and HP1α play roles in cancer growth, we assessed the effects of overexpressing these genes on lung cancer cell growth using stably transfected A549 lung cancer cells. We first used a clonogenic assay to assess the ability of single lung cancer cells to grow into colonies [54]. We found that, compared with control, A549 cells stably expressing STAT3-shRNA had more colonies, whereas cells expressing uSTAT3 (STAT3^Y705F^) or HP1α had fewer colonies (Fig. 4a, b). We then used the soft-agar assay [55] to assess the effects of uSTAT3 and HP1α on anchorage-independent growth of lung cancer cells. We found that knocking down STAT3 or HP1α in A549 cells by expressing shRNAi constructs resulted in more and larger colonies, whereas overexpressing uSTAT3 or HP1α led to fewer and smaller colonies in soft agar (Fig. 4c, d). These results are consistent with the notion that uSTAT3 and HP1α suppress cancer cell growth by increasing heterochromatin levels. The discrepancy for HP1α-shRNA expression in the two different assays may involve differences in cell survival and will be further investigated.

Finally, we investigated the effects of uSTAT3 and HP1α in cancer cell growth using mouse xenografts. We established lung cancer A549 cell lines stably expressing HP1α, STAT3^WT^, STAT3^Y705F^, and sh RNAi constructs targeting STAT3 and HP1α, respectively, as well as controls. We implanted these cells into immunodeficient (nude) mice subcutaneously and measured the growth of these cells as tumors in mice during the 4 weeks period after implantation. As shown at 4 weeks after implantation, we found that A549 cells expressing either HP1α or STAT3^Y705F^ exhibited reduced capacity to grow as tumors, as indicated by the small tumor volumes at week four compared with A549 cells expressing vector control (Fig. 4e). Conversely, knocking down HP1α or STAT3 by stably expressing RNAi constructs HP1α-sh or STAT3-sh resulted in larger tumor volumes (Fig. 4e). These results are consistent with a previous report that knocking down STAT3 in A549 cells promotes the growth of these lung cancer cells as mouse xenografts [38]. Taken together, our results suggest that uSTAT3 and HP1α play roles in tumor suppression.

## Discussion

We have previously discovered a noncanonical JAK/STAT pathway, in which uSTAT plays a role in promoting heterochromatin formation by associating with HP1 [1, 3, 4]. We have further shown that the noncanonical function of STAT is conserved from *Drosophila* to human STAT5A [2]. The physiological functions of this noncanonical STAT pathway and how many other STAT proteins have this function, however, remain incompletely understood. In this study, we used human lung cancer cells to investigate the non-canonical functions of STAT3, which has been implicated in many human cancers and has been reported to also have tumor suppressor function. We have found that STAT3 possesses a noncanonical function, is capable of associating with HP1, promoting heterochromatin formation, and suppressing tumor progression.

We have shown by immunostaining and FRET that uSTAT3 and HP1α colocalize, albeit partially, in the nucleus. The difference between uSTAT3 and uSTAT5A, which we have found to exhibit a more complete nuclear co-localization with HP1α [2], is intriguing and is still under investigation. Nonetheless, our studies of STAT3, and previously of STAT5A, suggest that the noncanonical function of uSTAT, initially identified in *Drosophila*, is conserved in mammals. These results are consistent with our previous report that knocking down HP1α enhances growth of tumor cells as mouse xenografts [2], and are consistent with reports that STAT3 functions as a tumor suppressor and loss of STAT3 promotes lung cancer cell growth in mouse xenografts [38]. Importantly, in this study we have shown that expressing uSTAT has effects opposite to knock downing STAT on cancer cell proliferation and tumor growth. Thus, our model that pSTAT promotes cancer development and uSTAT suppresses tumors by influencing heterochromatin dynamics can reconcile the contradicting results reported by different groups regarding the functions of STAT proteins, especially STAT3, in cancers.

Furthermore, how heterochromatin loss might lead to cancer development and how heterochromatin is initially established remain unclear at the molecular level [27, 28]. Noncoding RNA transcription is essential for heterochromatin formation in fission yeast [56, 57]. In other eukaryotes, however, DNA-binding proteins, including transcription factors, may initially bind to certain nucleation centers to recruit HMTs and/or HP1 [58–61]. Although BRCA1 and RB, and KLF11 can recruit HP1 to specific loci or maintain constitutive heterochromatin [58, 62–65], uSTAT recruiting HP1 to initiate new heterochromatin is yet to be investigated. It is conceivable that uSTAT is among the factors that can initiate heterochromatin formation by binding to specific DNA sequence motifs and recruiting HP1. Although several transcription factors have been implicated in heterochromatin formation across diverse species [58, 62–65], a prominent heterochromatin localization has only been shown for STAT proteins [1, 2]. Thus, STAT proteins might be among the protein factors that collectively or cooperatively control heterochromatin formation, which should have implications in both cancer biology and heterochromatin formation. The roles of uSTAT in heterochromatin initiation and maintenance and in cancer development are currently under investigation.

## Conclusions

We have shown that STAT3 possesses the non-canonical function by which uSTAT3 promotes heterochromatin formation. We propose that the tumor suppressor function of STAT3 is likely attributable to the heterochromatin-promoting activity of uSTAT3 in the non-canonical JAK/STAT pathway.

## Methods

### Source of cell lines

Human NSCLC cell lines A549, H226, H441, H460, and H520; and other human cancer cell lines HeLa and HEK293T were from American Type Culture Collection (ATCC, Manassas, VA 20110 USA).

### Cell culture, DNA constructs, and transfection

Human NSCLC cell lines A549, H226, H441, H460, and H520; and other human cancer cell lines HeLa and HEK293T cells were maintained in RPMI medium (Gibco) supplemented with 10% (v/v) FBS and antibiotics at 37 °C and with 5% CO_2_ in water-jacketed, humidified incubators. Transient transfection with plasmid DNA was done by using FuGENE 6 (Life Sciences, Inc) according to the manufacturer’s protocol. Cells stably transfected with the indicated cDNAs or shRNAs were selected as puromycin (5 μg/ml)-resistant colonies and several colonies were pooled. Cell culture and transfection procedure was approved by UCSD BUA R1347.

Plasmid DNA constructs with Myc-tagged human STAT3 and STAT3^Y705F^ were kindly provided by Dr. Pradipta Ghosh (UCSD). DNA construct for sh-HP1α was from Open BioSystems. The following DNA constructs were acquired from Addgene (Cambridge, MA): human HP1α (17652), sh-STAT3 (26596), and H3K9me3 FRET reporter (22866).

### Immunostaining, Immunoprecipitation, and Western blotting

For immunofluorescence, transfected cells were fixed in 4% paraformaldehyde for 10 min, permeabilized in 0.2% Triton X-100 in phosphate-buffered saline (PBS) for 15 min, blocked with 5% bovine serum albumin (BSA) in PBS for 30 min, and incubated overnight at 4 °C in primary antibodies. Primary antibodies used in this study include rabbit anti-STAT3 (1:250; Santa Cruz, sc-482), mouse anti-HP1 (CBX5) (1:200; Life Sciences, 730,019), goat anti-c-Myc (1:500, Fisher, NB600–335). Slides were washed four times in PBS and then immunostained with Alexa Fluor® 546-conjugated secondary antibody (1:250; Molecular Probes) at 37 °C for 1 h.

For co-immunoprecipitation, A549 cells were harvested in lysis buffer (50 mM Tris-HCl, pH 8.0, 250 mM NaCl, 5 mM EDTA, 1 mM DTT, 5% Glycerol) supplemented with complete protease inhibitor cocktail and PMSF at a final concentration of 2 mM. Portions of lysate containing equal amounts of protein (200 μg) were then immunoprecipitated overnight at 4 °C with protein G-Agarose (Roche) bound antibodies. The beads were then washed six times with lysis buffer, and associated protein complexes were recovered in SDS sample buffer. Protein samples were resolved on a 10% sodium dodecyl sulphate-polyacrylamide gel and transferred onto Pure Nitrocellulose Blotting Membranes (Pall) for immunoblot analysis. Rabbit anti-STAT3 (1:250; Santa Cruz, sc-482) and mouse anti-HP1 (CBX5) (1:200; Life Sciences, 730,019) primary antibodies were used to detect endogenous HP1α and STAT3, respectively, followed by incubation with horseradish peroxidase (HRP) conjugated secondary antibodies and visualization using an enhanced chemiluminescence kit (Pierce). Full-length gel images are shown in **Fig. S1**.

### Fluorescence resonance energy transfer (FRET)

To detect changes in histone methylation (H3K9me3) levels in living cells, cells were transfected with a CFP/YFP histone methylation FRET reporter construct [48]. Donor and acceptor bleed through was corrected using donor and acceptor only samples. FRET measurements were carried out using a Zeiss Axio Observer fluorescence microscope equipped with FRET setup and software.

To detect FRET in fixed cells, cells were fixed on a coverslip and immunostained with anti-HP1α-Alexa488 (donor) to anti-STAT3-Alexa546 (acceptor) primary and secondary antibody pairs, as described in [47]. Donor and acceptor bleed through was corrected using donor and acceptor only samples. FRET was detected and processed using a Leica confocal microscope with built-in FRET software.

### Senescence-associated beta-galactosidase assay

Cells were plated on 24 well plates. Nearly confluent cells were fixed with 3.7% Paraformaldehyde in PBS. 500ul of β-gal staining solution (0.1% X-Gal, 5 mM 5 mM potassium ferrocyanide, 5 mM potassium ferricyanide, 150 mM Sodium chloride, and 2 mM Magnesium chloride in 40 mM citric acid/sodium phosphate solution, pH 6.0) was added and plates were incubated at 37 °C overnight. After removal of staining solution, 70% glycerol was added and visualized with 20X bright field microscope. Senescence cells show blue staining in the cytosol [53].

### Quantitative PCR measurement of major satellite transcripts

Total RNA from A549 cells stably expressing the indicated transgene was isolated using RNeasy Plus Mini kit (Qiagen) according to the manufacturer’s manual. The SuperScript IV First-Strand Synthesis System (Thermo Fisher) was used to generate cDNA, according to the manufacturer’s manual, and was subjected to Sybr Green qPCR using the Applied Biosystems 7300 Real Time PCR instrument per manufacturer’s protocol. Major satellite transcripts expression values were normalized relative to *Gapdh*. Primers used for qPCR are as the following.

Major Sattellite Forward: AGGGAATGTCTTCCCATAAAAACT.

Major Satellite Reverse: GTCTACCTTTTATTTCAATTCCCG.

Gapdh forward: CATGGGTGTGAACCATGAGA.

Gapdh reverse: CAGTGATGGCATGGACTGTG.

### Anchorage-independent growth (soft agar) assay

Stable A549 cell lines with the indicated transgene were maintained in RPMI medium supplemented with 10% fetal bovine serum. Cells grown to 60% confluency were resuspended in 0.4% Noble agar (Sigma, St. Louis, MO) in RPMI, and were seeded at a density of 1.5 × 10^5^ cells/well in 6-well culture plates on top of a 2 ml underlayer composed of 0.8% agarose in RPMI. Media were refreshed twice per week for 3 weeks, and then were stained with p-iodonitrotetrazolium violet (Sigma, St. Louis, MO) and photographed on an inverted compound microscope with phase contrast optics.

### Colony formation assay

Stable A549 cell lines with the indicated transgene were maintained in RPMI medium supplemented with 10% fetal bovine serum. Cells grown to 60% confluency were harvested and diluted in fresh media, and seeded at 100 cells/plate in 6-well plates. Cells were maintained in RPMI medium (Gibco) supplemented with 10% (v/v) FBS and antibiotics at 37 °C and with 5% CO_2_ in a water-jacketed, humidified incubator until large colonies (in excess of 50 cells) were visible in control plates. Medium was removed and cells were fixed with a solution of 6% glutaraldehyde and 0.5% crystal violet in PBS, and the number of colonies was counted.

### Xenograft assays

Tumor formation was assayed by xeno-implantation of genetically perturbed cells. Prior to xeno-implantation, transfected cells were grown for 48 h under standard culture conditions without selective drugs. 5 × 10^5^ cells were subcutaneously injected into the left and right flanks of 4–6 month old female CD-1 nude mice (Crl:CD-1-Foxn1^nu^, Charles River Laboratories). Tumor volumes were measured weekly using a caliper for 4 weeks. Following final tumor measurements, mice were euthanized by CO2 inhalation per IACUC protocol. Tumor volume was calculated using the average of 3 measurements of the tumor radius and the formula Volume = (4/3)πr^3^. The statistical significance of differences in tumor size was determined by Student’s *t*-test. The vertebrate animal protocol has been approved by UCSD Institutional Animal Care and Use Committee (IACUC) (Protocol Number: S13282).

## Supplementary information

**Additional file 1 Supplementary Figure S1.** Full-length gel images for Figure 1B, 2A.

## Data Availability

• All data generated or analysed during this study are included in this published article [and its supplementary information files].

## Abbreviations

FRET: Fluorescence Resonance Energy Transfer
HP1: Heterochromatin Protein 1
JAK: Janus kinase
NSCLC: Non-small cell lung cancer
PSTAT: Phosphorylated STAT
QRT-PCR: Quantitative real-time polymerase chain reaction
SA-βgal: Senescence-associated β-galactosidase
STAT: Signal Transducer and Activator of Transcription
USTAT: Unphosphorylated STAT
