# Supplementary figures and images for: Unphosphorylated STAT3 in heterochromatin formation and tumor suppression in lung cancer
Source: BMC Cancer. 2020 Feb 22;20:145. doi: 10.1186/s12885-020-6649-2 (PMC7036253; doi:10.1186/s12885-020-6649-2)

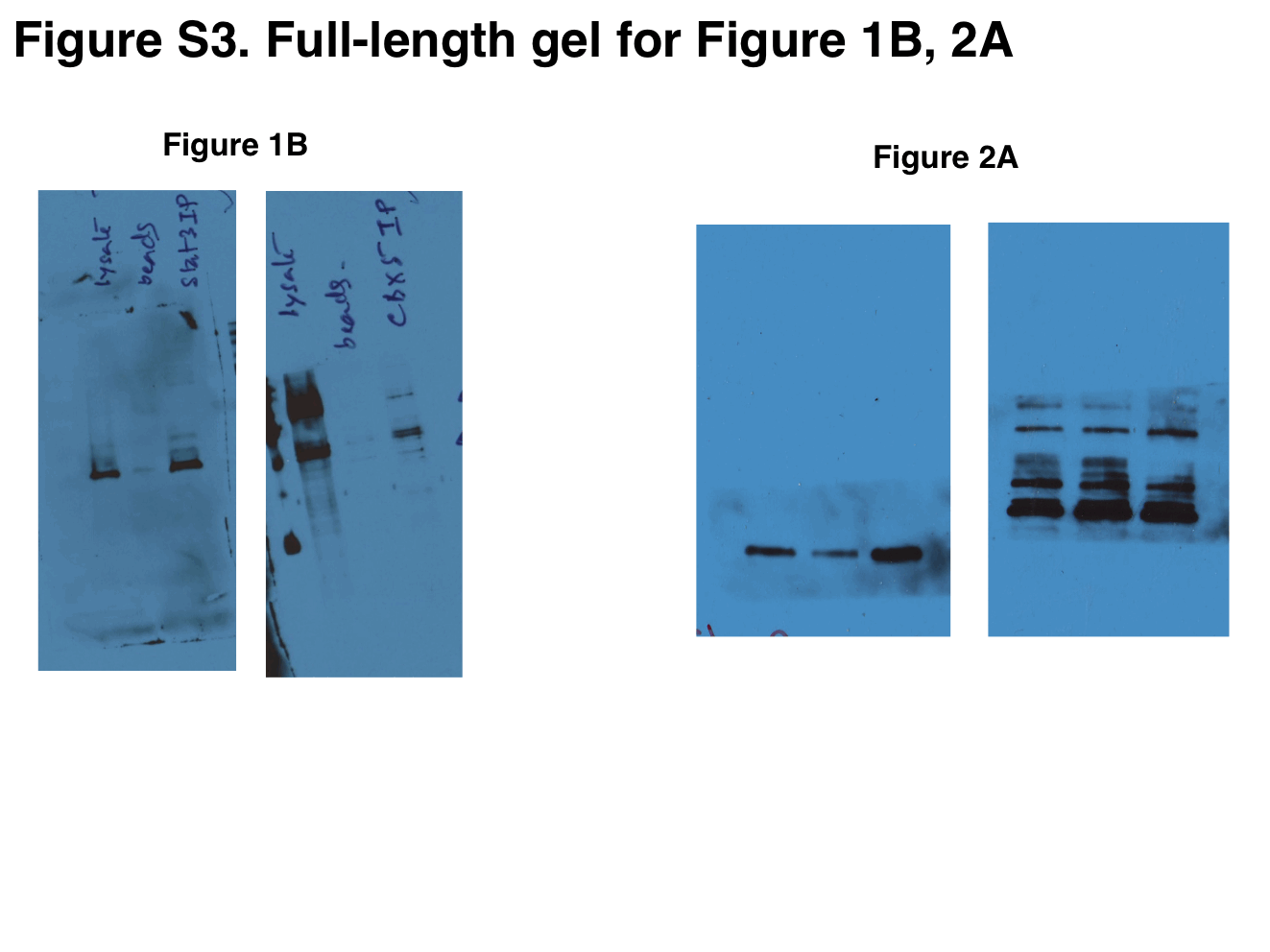

Supplement: Supplementary file 1 — Additional file 1 Supplementary Figure S1. Full-length gel images for Figure 1B, 2A. [file 12885_2020_6649_MOESM1_ESM.gif]
